# Guaiazulene Triggers ROS-Induced Apoptosis and Protective Autophagy in Non-small Cell Lung Cancer

**DOI:** 10.3389/fphar.2021.621181

**Published:** 2021-04-15

**Authors:** Qin Ye, Li Zhou, Ping Jin, Lei Li, Shuwen Zheng, Zhao Huang, Jiayang Liu, Siyuan Qin, Hao Liu, Bingwen Zou, Ke Xie

**Affiliations:** ^1^ Department of Oncology, Sichuan Academy of Medical Sciences and Sichuan Provincial People’s Hospital, School of Medicine, University of Electronic Science and Technology of China, Chengdu, China; ^2^ State Key Laboratory of Biotherapy and Cancer Center, West China Hospital, West China School of Basic Sciences and Forensic Medicine, Collaborative Innovation Center for Biotherapy, Sichuan University, Chengdu, China; ^3^ School of Basic Medical Sciences, Chengdu University of Traditional Chinese Medicine, Chengdu, China; ^4^ Department of Thoracic Oncology and Department of Radiation Oncology, Cancer center, West China Hospital, Sichuan University, Chengdu, China

**Keywords:** guaiazulene, autophagy, non-small cell lung cancer, AKT/mTOR, apoptosis

## Abstract

Non-small cell lung cancer (NSCLC) is one of the most frequent cancers worldwide, yet effective treatment remains a clinical challenge. Guaiazulene (GYZ), a cosmetic color additive, has previously been characterized as a potential antitumor agent due to observed anticancer effects. However, the efficacy of GYZ in the treatment of NSCLC and the involved molecular mechanisms remain largely unknown. Here, we indicated a role for GYZ in the suppression of NSCLC both *in vitro* and *in vivo* via triggering reactive oxygen species (ROS)-induced apoptosis*.* Concomitantly, GYZ induced complete autophagic flux in NSCLC cells via inhibiting the Akt/mTOR signaling pathway, which displayed cytoprotective effect against GYZ-induced growth suppression. Accompanied with autophagy inhibition obviously enhanced the effects of GYZ. Notably, GYZ acts synergistically with paclitaxel in the suppression of NSCLC *in vitro*. Together, our results for the first time reported that GYZ suppressed the proliferation of NSCLC and suggested a potential strategy for inhibiting NSCLC growth by combinational use of GYZ and autophagy inhibitors.

## Introduction

Lung cancers are the leading cause of cancer incidence and mortality with nearly 2.1 million new lung cancer cases diagnosed and over 1.8 million deaths worldwide (Bray et al., 2018). Lung cancers are mainly classified as non-small cell lung cancer (NSCLC) and small cell lung cancer (SCLC), among which NSCLC accounts for about 85% of all lung cancers (Zhang R. et al., 2019). Surgical resection, in combination with radiotherapy or/and platinum-based chemotherapy when necessary, affords curative treatment for early or local disease. However, many patients with advanced lung cancer require chemotherapy, target therapy, immunotherapy, or combinations of these (Aggarwal and Borghaei, 2017; Shaw et al., 2019; Ramalingam et al., 2020). Despite the diverse treatment strategies have been proposed to improve the prognosis of patients, resistance and relapse are extremely common (Clara et al., 2019). Thus, there is an urgent need to develop novel potential therapeutic agents for lung cancer treatment.

Guaiazulene (1,4-dimethyl-7-isopropylazulene, GYZ), a dark blue crystalline hydrocarbon, is mainly extracted from the oil of *Guajacum ojficinale* tree and *Matricaria chumomilla* tree distributed in northern coast of the Caribbean and South America (Kourounakis et al., 1997a; Gunes et al., 2013). GYZ has been reported to display many beneficial biological activities, such as anti-oxidant, anti-inflammatory, anti-ulcer or used as a cosmetic color additive (Yanagisawa et al., 1990; Kourounakis et al., 1997a; Guarrera et al., 2001; Wang et al., 2003). In addition, GYZ showed considerable anticancer properties in human oral squamous cell carcinoma (OSCC) (Wakabayashi et al., 2003; Uehara et al., 2018), neuroblastoma (NB) (Togar et al., 2015) and human promyelocytic leukemia (Pratsinis and Haroutounian, 2002). However, to date limited data exist indicating the efficacy of GYZ in the treatment of NSCLC and investigating the potential mechanisms.

Apoptosis is a widely known fundamental biological process that occurs via the classic intrinsic or extrinsic signaling pathway, triggering and regulating by many different molecular pathways (Pritchard and Watson, 1996; Qiao and Wong, 2009). One of the distinct cell death subroutine of caspase-dependent apoptosis is known as anoikis (Vachon, 2018). Anoikis is a kind of programmed cell death which prevents cell attachment to an inappropriate matrix and adherent-independent cell growth, thus avoiding colonizing of distant organs (Paoli et al., 2013). This special pattern of cell death is mainly caused by the decrease of integrins (Frisch and Ruoslahti, 1997). Integrins containing integrin β1 and β3 modulate cell signaling, adhesion, and survival through transmitting extracellular signals across the plasma membrane (Alanko et al., 2015). Loss of the interaction between integrins, extracellular matrix (ECM) and cellular adhesive molecules leads to the deficiency of survival signals in non-adherent cells, followed by induction of apoptotic cell death (Vachon, 2011). Previously studies have shown that Caveolin-1 plays a role on deprivation of anoikis response in non-small cell lung cancer (Chanvorachote et al., 2015; Li et al., 2019). Moreover, the down-regulation of anti-apoptosis Bcl-2 family proteins including Mcl-1 (myeloid cell leukemia 1) and Bcl-2 (B-cell lymphoma 2) have been proved to associate with anoikis (Pongrakhananon et al., 2010; Chunhacha et al., 2012). Numerous studies have indicated that anti-tumor drugs suppress tumor progression by inducing cell anoikis. For example, Tetrathiomolybdate has been found to promote head and neck cancer cell anoikis, thus inhibiting tumor cell metastasis (Kumar et al., 2010). Additionally, another study showed that Avicequinone B triggered anoikis in non-small cell lung cancer through down-regulation of integrin-mediated survival signaling and anti-apoptosis proteins (Prateep et al., 2018). However, there is limited evidence indicating whether GYZ induces cancer cells anoikis so far.

Autophagy is a process of degradation which is to deliver cytosolic materials to the interior of the lysosomes, in order to recover sources of requisite metabolites and metabolic energy in times of energy limitation, starvation and hypoxia (Green and Levine, 2014). Previous studies have indicated that there are four main functional forms of autophagy, including cytoprotective, nonprotective, cytostatic and cytotoxic autophagy in tumor cells in the context of chemotherapy or radiation (Gewirtz, 2014). For example, one study revealed a potential mechanism of Paclitaxel resistance in NSCLC. It indicated that Paclitaxel upregulated Beclin-1 via decreasing miR-216b levels, which induces cytoprotective autophagy in NSCLC cells, thus antagonizing paclitaxel-induced cell death (Chen and Shi, 2016). In addition, another study showed that cotreatment with Cisplatin and Bu-Zhong-Yi-Qi decoction (BZYQD), a Chinese traditional herbal medicine, would activate apoptosis and autophagy by oxidative stress, thus enhancing the antitumor effect (Yu et al., 2017). Therefore, the role of autophagy in determining NSCLC cell fate is complex. Figuring out the biologic function of autophagy in response to NSCLC anticancer therapy is scientifically worthwhile for providing potential novel therapeutic strategies.

In this study, we found that GYZ induced NSCLC cell death both *in vitro* and *in vivo*. GYZ induced mitochondrial dysfunction, and thereby inhibited tumor growth in NSCLC. GYZ also promoted remarkable autophagosome synthesis and the fusion of autophagosomes and autolysosomes, resulting from the blockade of the Akt/mTOR signaling. This complete autophagic flux displayed cytoprotective role for NSCLC cells, thus impairing the anticancer effect of GYZ. Accordantly, GYZ treatment accompanied with autophagy inhibition obviously enhanced the growth suppression of GYZ on NSCLC cells. Notably, GYZ acted synergistically with paclitaxel in the suppression of NSCLC *in vitro*. Together, our results provide a novel link between GYZ-induced growth suppression and cytoprotective autophagy. Targeting cytoprotective autophagy with GYZ treatment may hold the promise for suppressing NSCLC growth.

## Materials and Methods

### Cell Culture and Reagents

Human non-small lung cancer cell lines A549, H1975, HCC827, PC9, H1299 were purchased from the American type culture collection (ATCC). Cells were maintained in RPMI1640 supplemented with 100 U/ml penicillin (Millipore Sigma), 100 mg/ml streptomycin (Millipore Sigma), and 10% fetal bovine serum (Gibco), in a humidified incubator at 37°C under 5% CO_2_ atmosphere.

Reagents used were as follows: 3-Methyladenine (HY-19312), Paclitaxel (HY-B0015), Cisplatin (DDP) (HY-17394) and Z-VAD-FMK (HY-16658B) were purchased from MedChem Express (MCE). Chloroquine diphosphate salt (C6628) was purchased from Sigma. Guaiazulene (GYZ) (G137246) was purchased from Aladdin. GYZ, Paclitaxel were dissolved in DMSO. 3-methyladenine (3MA), and chloroquine diphosphate salt (CQ) were dissolved in phosphate-buffered saline (PBS). DDP was dissolved in N, N-Dimethylformamide (DMF).

Antibodies used in this study: Cleaved-caspase 3 (ZEN BIO, 380189), Caspase 3 (ZEN BIO, 380189), PARP (Cell Signaling Technology, 9532), Cleaved-PARP (Cell Signaling Technology, 9532), Akt (Cell Signaling Technology, 4685), phosphorylated (p-)Akt (Ser473) (Cell Signaling Technology, 4060), mTOR (Cell Signaling Technology, 2972), p-mTOR (Ser2448) (Cell Signaling Technology, 2971), p70S6K (Cell Signaling Technology, 9202), p-p70S6K (Ser371) (Cell Signaling Technology, 9208), 4EBP1 (Cell Signaling Technology, 9452), p-4EBP1 (Ser65) (Cell Signaling Technology, 9451), β-actin (Santa Cruz, sc-1616), Ki67 (Abcam, ab66155), LC3 (Novus, NB100-2220), horseradish peroxidase (HRP)-conjugated anti-mouse secondary antibody (Santa Cruz, sc-2005), horseradish peroxidase (HRP)-conjugated anti-rabbit secondary antibody (Santa Cruz, sc-2004). For immunofluorescence, goat anti-rabbit Alexa Fluor 488, goat anti-mouse Alexa Fluor 594 were obtained from Thermo Fisher Scientific.

### Cell Growth and Proliferation Assays

The short-term effects of GYZ on NSCLC cell proliferation were detected using the thiazolyl blue tetrazolium bromide (MTT) (Sigma, M2128) assay. Seed cells in a 96-well plate (5,000 cells/well). After 48 h treatment with the indicated concentrations of GYZ, MTT were added and incubated for 2–4 h. Then removed the medium and added 100ul DMSO to each well. The absorbance was determined at 570 nm with ELISA multiwell spectrophotometer.

Colony formation assay was used to examine the long-term effects of GYZ on NSCLC cell growth. Seed cells in a 24-well plate (200 cells/well) for about 7 days, treated with the indicated concentration of GYZ for 36–48 h. After 2 weeks, the colonies were fixed with 4% paraformaldehyde (Sigma, 16005) for 30 min and washed three times. Then stained with crystal violet for another 30 min. After washing for three times, the visible colonies were taken photos and counted using ImageJ software.

### Detachment-Induced Cell Death and CCK8 Assay

In order to prevent anchorage, human non-small cell lung cancer cells were seeded in an ultra-low attachment plate. A single-cell suspension of non-small cell lung cancer cells at a density of 5,000 cells/well was maintained in RPMI1640 culture medium. After treatment with the indicated concentration of GYZ for 48 h, the cells were evaluated of cell viability through the incubation with 10 μM CCK-8 (Beyotime Biotechnology, C0038) at 37°C for 40 min. The absorbance value was then detected at 450 nm.

### Intracellular ATP Detection Assay

The intracellular ATP was detected using a ATP detection kit (Beyotime Biotechnology, S0155S). Seed cells in a 96-well plate (5,000 cells/well). After 48 h treatment with the indicated concentrations of GYZ, discarded the culture medium. Then added 30 μl ATP detection lysate to each well and placed it on ice for 30 min until the cells were fully lyzed. In a new 96-well plate, add ATP detection working solution (50 μl/well). After inoculating at room temperature for 5 min, lyzed cell supernatant (10 μl/well) was added to the ATP detection working solution, mix quickly, measure the RLU value with a microplate reader, and analyze the relative concentration of ATP under different processing conditions.

### Lactate Dehydrogenase Release Assay

The cytotoxicity of GYZ was analyzed by Lactate dehydrogenase release assay using LDH Cytotoxicity Assay Kit (Beyotime Biotechnology). Cells were seeded in a 96-well plate at a density of 10,000 cells/well and treated with GYZ at the indicated concentrations. The negative control was cultured in medium without GYZ treating. After 48 h treatment, the 96-well plate was centrifuged at 500 *g* for 3 min. Eighty microlitres of supernatant from each well was transferred to a new 96-well plate and 40 μl of LDH maximum leakage (according to the manufacturer’s instructions) was added too, following centrifugation. After the 96-well plate was prevented from light for 30 min, the absorbance value was then detected at 492 nm or 570 nm with ELISA multiwell spectrophotometer.

### Tumor Xenograft Animal Models

All animal studies were approved by the Institutional Animal Care and Treatment Committee of Sichuan University. Five-week-old female nude mice (BALB/c, 17–19g each) were purchased from HFK Bioscience Co., Ltd. (Beijing). The mice were housed under standard conditions. For establishment of xenograft model, A549 cells (5 × 10^6^ cells/mouse) were suspended in PBS and subcutaneously injected into flanks of mice. When the tumor volume reached 50–100 mm^3^ (about 10 days post-injection), mice were randomly grouped and intraperitoneally injected with 100 μl of vehicle (5% DMSO, 5% Ricinus oil, 90% physiological saline), GYZ (25 mg/kg/day). The mice weight and tumor volumes were measured every day (tumor volume (mm^3^) = (length × width^2^)/2). After 3 weeks, the mice were euthanized. The major organs (heart, lung, liver, spleen, kidney) and tumors were harvested.

### Immunoblotting

Cells were lyzed with RIPA buffer (150 mM NaCl, 50 mM Tris base, 1.0 mM EDTA, 1% sodium deoxycholate, 0.1% SDS, 1% Triton X-100, 1 mM PMSF) supplemented with phosphatase and protease inhibitor cocktail (Thermo fisher scientific, 23250). Then, protein lysates were centrifuged (15,000r, 10 min) and boiled with loading buffer. Next, SDS-PAGE was used to separate protein, and then transferred protein onto PVDF membranes. Followed by blocking with skimmed milk (5%) in 1*TBST. After the primary and secondary antibodies was incubated, the ECL (EMD Millipore, WBKLS0500) was used to examine the immunoreactivity.

### EdU Labeling Assay

The 5-ethynyl-20-deoxyuridine (EdU) labeling assay was performed in 96-well plates at a density of 5000 cells/well using the EdU Cell Proliferation Assay Kit (Ribobio). After 48 h of GYZ treatment, 10 μM EdU was added to each well. Followed by incubating for another 24 h at 37°C, Cells were then fixed with 4% paraformaldehyde and stained with reaction cocktail. Next, DAPI was used for nuclear staining, followed by imaging with a fluorescence microscope (Leica).

### Flow Cytometry

Apoptosis analysis was performed using the annexin V–FITC/propidium iodide (PI) Detection Kit (KeyGen Biotech, KGA108) (following the manufacturer’s protocol). Cells were harvested and washed three times with cold PBS. After cells were resuspended in 500 μl binding buffer, 5 μl PI and 5 μl annexin V-FITC were both added into the cell suspension. At least 10,000 live cells were analyzed on a FACSCalibur flow cytometer (BD Biosciences). FlowJo software was used to analyze the data.

Intracellular ROS content was detected using the fluorescent probe DCFH-DA. Inoculate an appropriate amount of cells in a six-well plate, and treat them with GYZ for 48 h after adherence. Replace the old medium with double-free medium, add 5 μM DCFH-DA to each well, and place it in the cell culture incubator for 30 min. Cells were washed with cold PBS for three times. After cells were resuspended, FACSCalibur flow cytometer (BD Biosciences) was used to measure the fluorescence intensity. FlowJo software was used to analyze the data.

### Determination of Mitochondrial Superoxide by Mitosox

Cells were plated on glass cover slips at a density of 25,000 cells/well in 24-well plates. After 48 h of GYZ treatment at the indicated concentrations, discard the culture medium and wash twice with sterile PBS. Ten micrometer Mitosox (Thermo, M36008) PBS solution was added to each well. After incubation in the cell incubator for 30 min, discard the working solution, wash twice with sterile PBS again. Finally, put the glass cover slips upside down on the glass slide, mount the slides with glycerol and observe the snapshots under an upright fluorescence microscope.

### Immunohistochemistry

Immunohistochemical staining was measured as described previously (Jin et al., 2019). All stained sections were viewed with a DM2500 fluorescence microscope. Four grades (0, negative; 1, weak; 2, moderate; 3, strong) of the immunostaining intensity (A) and the proportion of positive cells (B) (0–100%) were evaluated the immunohistochemical staining. The final score was calculated by multiplying the A and B.

### Immunofluorescence

Cells were seeded onto the glass cover slips at the density of 20,000 cells/well in 24-well plates. After 48 h of GYZ treatment, cells were fixed in 4% paraformaldehyde for 30 min. Next, the slides were washed with PBS and permeabilized with 0.4% Triton X-100 and blocked with 5% fetal bovine serum for 1 h. After being incubated with primary antibody overnight at 4°C, cells were incubated with secondary antibody (DyLight 594–conjugated goat anti-mouse IgG or DyLight 488–conjugated goat anti-rabbit IgG) at 37°C for 1 h. Finally, nuclei were stained with DAPI for 8 min. Images were captured using a confocal laser scanning microscopy (Carl Zeiss). The number of LC3 puncta per cell was countered by the software “Image J”, the area size represents relative numbers.

### Statistical Analysis

Statistical analysis and graphics were measured using GraphPad 7.0 software. Two-way ANOVA and Student *t*-test were used to analyze statistical differences. Grayscale statistics were measured by Image J software. Data were presented as means ± SD from at least three individual experiments. Statistically significance was described as follows: *, *p* < 0.05; **, *p* < 0.01; ***, *p* < 0.001.

## Results

### GYZ Inhibits the Growth of NSCLC Cells *In Vitro*

**FIGURE 1:**
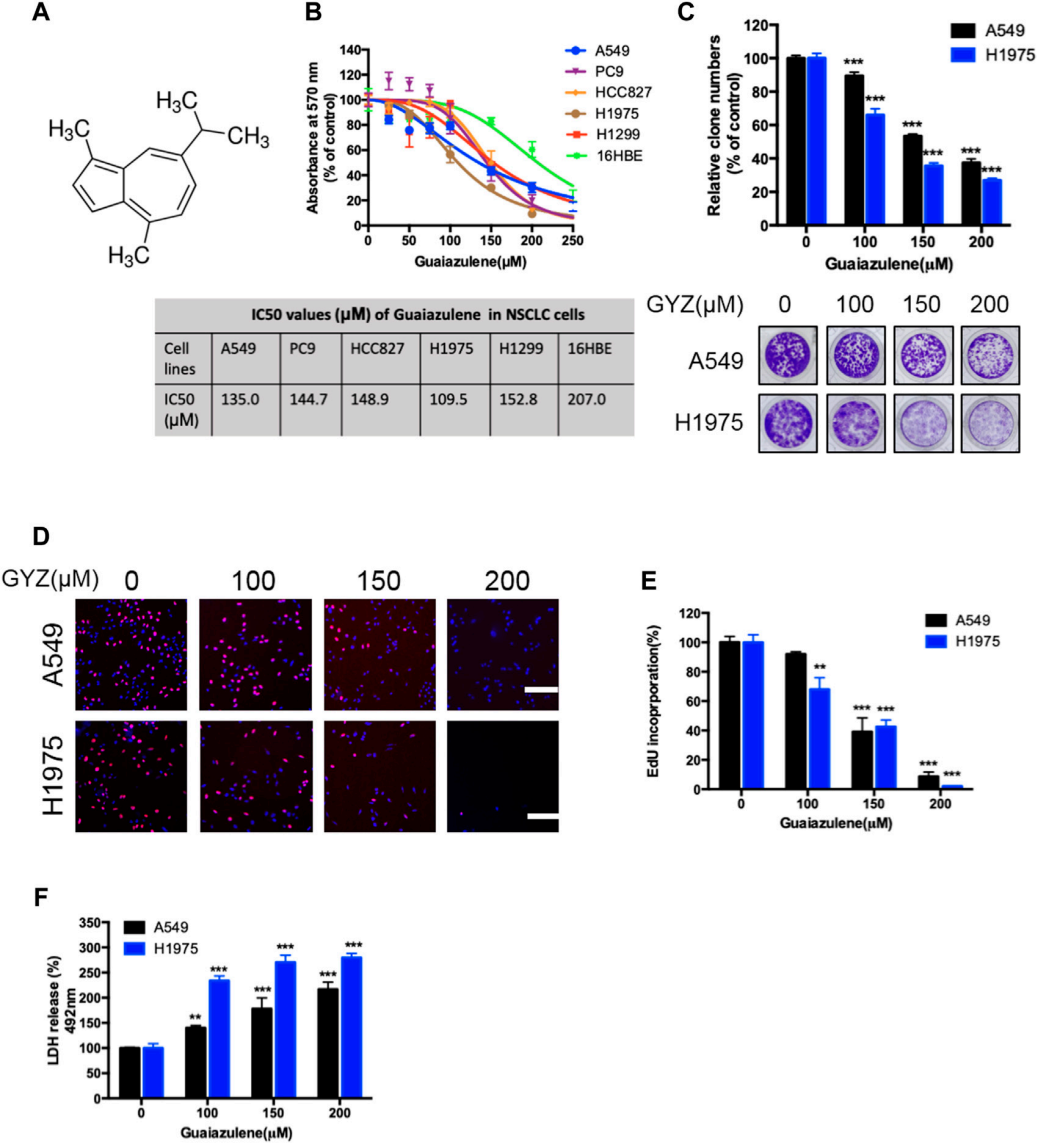
GYZ inhibits the growth of NSCLC cells *in vitro*. **(A)** Chemical structure of GYZ. **(B)** NSCLC cells were treated with GYZ at the indicated concentrations for 48 h, and cell growth was measured by MTT assay. **(C–E)** A549 cells and H1975 cells were treated with GYZ at the indicated concentrations (the tested doses of guaiazulene were determined by the IC50 values). Cell proliferation was performed by colony formation **(C)** and EdU incorporation **(D,E)** assays. **(F)** Cells were subjected to GYZ for 48 h, and cytotoxicity was examined by the release of LDH.

To validate whether GYZ exhibits an anticancer effect against NSCLC, we first detected the cell growth in response to GYZ treatment in various human NSCLC cell lines. MTT assay showed that GYZ treatment for 48 h markedly decreased the cell viability of different NSCLC cell lines (A549, PC9, HCC827, H1975 and H1299) in a dose-dependent manner, while the median inhibitory concentration (IC50) value in 16HBE (a kind of immortalized epithelial cell from human respiratory tract) cells was higher than those in NSCLC cells (Figures 1A,B). Concomitantly, the proliferation of NSCLC cells was obviously inhibited under GYZ treatment, as evidenced by reduced colony numbers (Figure 1C). Furthermore, we performed EdU labeling assay and found that in comparison to controls, a significantly lower rate of EdU-positive cells was observed in GYZ-treated cells (Figures 1D,E). LDH release assay revealed that GYZ treatment showed marked cytotoxicity in A549 and H1975 cells (Figure 1F). Collectively, these results demonstrate that GYZ inhibits the growth of NSCLC cells *in vitro*.

### GYZ Triggers Apoptosis in NSCLC Cells *In Vitro*

**FIGURE 2:**
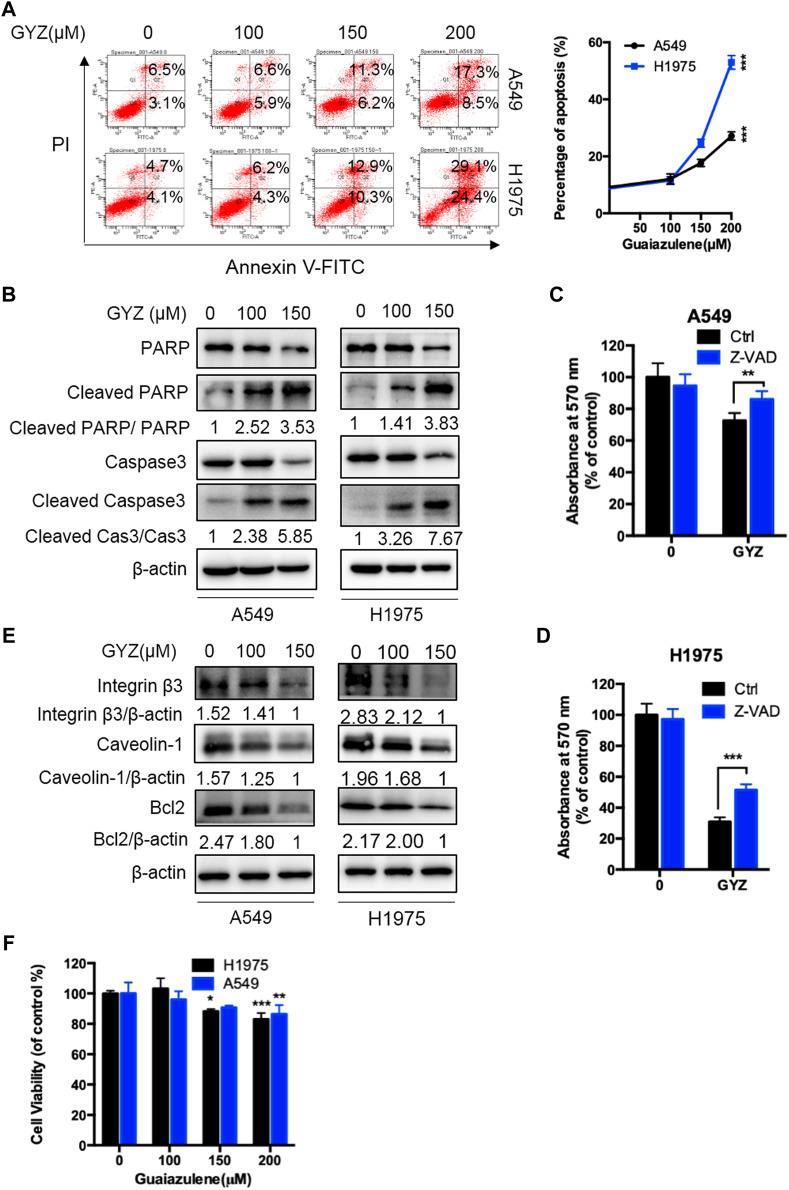
GYZ triggers apoptosis in NSCLC cells. **(A)** annexin V–FITC/PI staining was used to detect the apoptotic effects induced by GYZ in NSCLC cells. **(B)** Immunoblot analysis of Cleaved PARP, PARP, Cleaved Caspase3, Caspase3 expression in NSCLC cells treated with the indicated concentrations of GYZ for 48 h. **(C,D)** The MTT assay determined cell growth of NSCLC cells treated with GYZ (150 μM) in the absence or presence of 10 μM Z-VAD for 48 h. **(E)** Immunoblot analysis of Integrin β3, Caveolin-1, Bcl2 expression in NSCLC cells treated with the indicated concentrations of GYZ for 48 h. **(F)** GYZ at the indicated concentrations induced anoikis in NSCLC cells that were cultured under detachment environment for 24 h. All data are means ± SD. *p < 0.05, **p < 0.01, ***p < 0.001.

To examine whether GYZ induces apoptosis in NSCLC cells, we evaluated the apoptotic rate in GYZ-treated cells and control cells through flow cytometry assays. As shown in Figure 2A, GYZ treatment for 48 h showed significantly apoptosis induction in A549 and H1975 cells. Concomitantly, this was further evidented by the increased level of cleaved-caspase 3 and cleaved-PARP in GYZ-treated NSCLC cells (Figure 2B). Furthermore, GYZ treatment accompanied with a well-known pan caspase inhibitor Z-VAD-FMK was performed. The combinatorial treatment restored GYZ-induced growth suppression, as evidenced by restored cell growth in combinatorial treatment cells (Figures 2C,D). Anoikis is kind of apoptotic cell death which is induced by detachment condition. Integrins played the central role in suppressing anoikis by promoting survival signals and eliciting anti-apoptotic from the ECM (Giancotti and Ruoslahti, 1999). Many studies have also reported that Caveolin-1 and anti-apoptosis Bcl-2 family proteins including Bcl-2 are associated with anoikis (Ravid et al., 2005; Pongrakhananon et al., 2010). To test whether GYZ induces anoikis in NSCLC cells, we found that the protein level of Integrin β3, Bcl-2 and Caveloin-1 were obviously decreased after GYZ treatment in A549 and H1975 cells (Figure 2E). This was further supported by CCK8 assay during which A549 and H1975 cells were in non-adherent circumstance. As shown in Figure 2F, after GYZ treatment at the indicated concentrations, the reduction of cell survival was detected. Together, our data suggest that treatment of GYZ displays an obvious antiproliferative effect on NSCLC cells partially by induction of anoikis.

### ROS Accumulation Are Responsible for Anti-NSCLC Effects of GYZ

**FIGURE 3:**
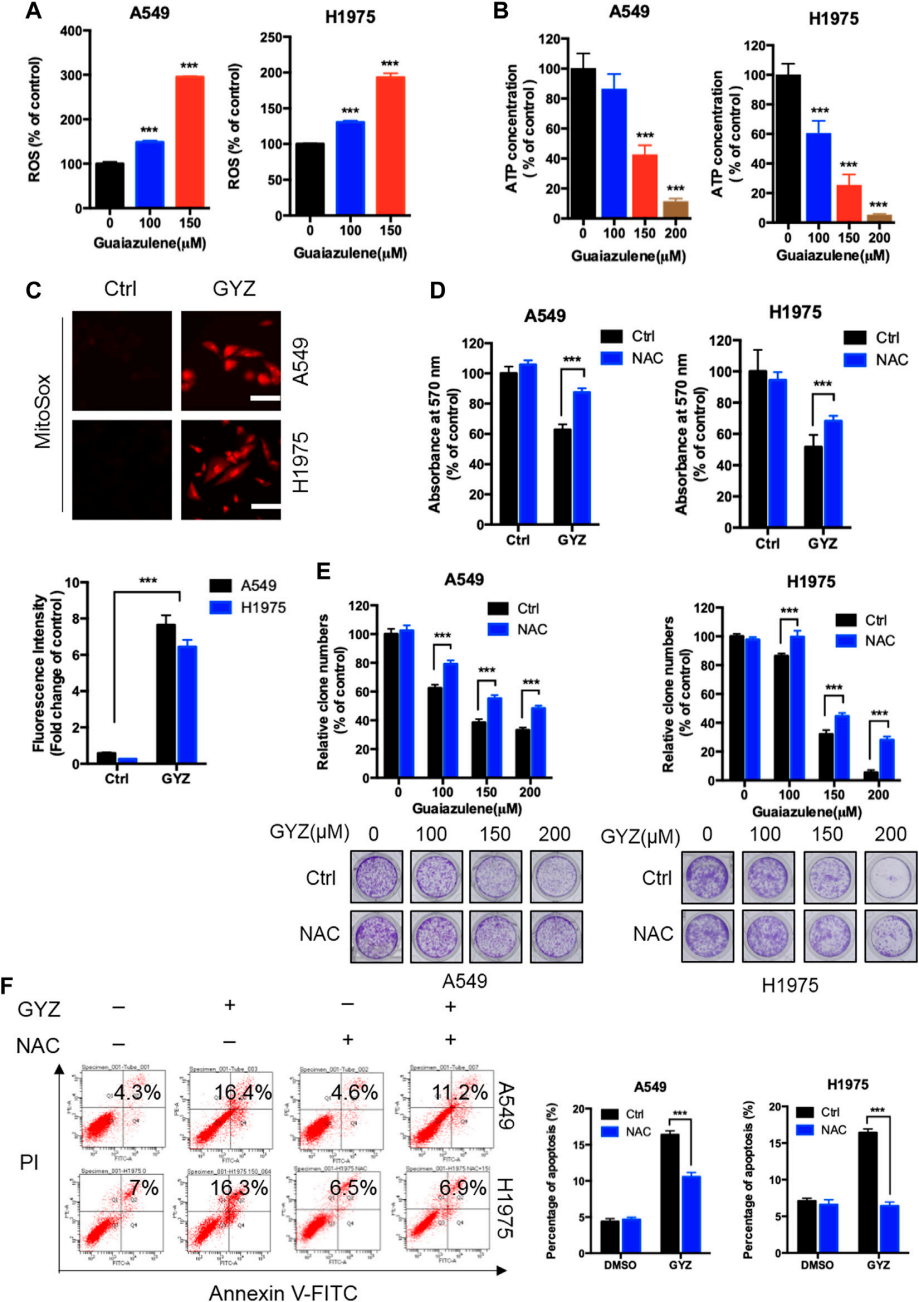
ROS accumulation are responsible for anti-NSCLC effects of GYZ *in vitro*. **(A)** ROS level was measured by DCFH-DA staining through a fluorescence microplate Reader in NSCLC cells treated with the indicated concentrations of GYZ. **(B)** Mitochondrial mass analyzed by ATP content measurement in A549 and H1975 cells treated with the indicated concentrations of GYZ. **(C)** Mitochondrial ROS accumulation was detected using MitoSOX Red in NSCLC cells treated with or without GYZ. **(D)** The MTT assay determined cell viability of NSCLC cells treated with GYZ (100 μM) in the absence or presence of 2 mM NAC for 48 h. **(E)** Colony formation assay of NSCLC cells treated with the indicated concentrations of GYZ in the absence or presence of 2 mM NAC. **(F)** Annexin V–FITC/PI staining was used to detect the apoptotic effects induced by GYZ (150 μM) in the absence or presence of 2 mM NAC for 48 h in NSCLC cells. Scale bars, 25 μm. All data are means ± SD. *p < 0.05, **p < 0.01, ***p < 0.001.

Increasing papers have shown that reactive oxygen species (ROS) is a critical mediator for the regulation of cell death of cancer cells (Zhou et al., 2014; Jiang et al., 2015). To detect whether GYZ induces NSCLC cell death via increasing cellular ROS levels, we performed flow cytometry assay by DCFH-DA staining via a fluorescence microplate reader in GYZ-treated cells and control cells. As shown in Figure 3A, GYZ treatment dramatically increased cellular ROS levels. As the mitochondrial respiratory chain is one of the main cellular sources of ROS (Sarniak et al., 2016), we thus measured the function of mitochondrial in GYZ-treated NSCLC cells. In line with the accumulation of ROS levels, the mitochondrial function was aberrant under GYZ treatment as evidenced by decreased ATP level (Figure 3B). In addition, we also measured mitochondrial ROS generation to indicate the functional alterations. Collectively, we found that mitochondria in GYZ-treated cells produced more superoxide than control cells (Figure 3C). Next, to identify the role of ROS in GYZ-induced growth suppression, GYZ treatment accompanied with ROS scavenger N-Acetyl-cysteine (NAC) was performed. The combinatorial treatment restored GYZ-induced growth suppression, as evidenced by restored cell viability and colony formation in combinatorial treatment cells (Figures 3D,E). Moreover, to further demonstrate the involvement of ROS in apoptosis, we evaluated the apoptotic rate after the combinatorial treatment of NAC through flow cytometry assays. As shown in Figure 3F, NAC could attenuate guaiazulene-induced apoptosis in A549 and H1975 cells. Together, these results demonstrate that GYZ induces cellular ROS accumulation, thereby inducing NSCLC cell apoptosis.

### GYZ Stimulates Autophagy in NSCLC Cells

**FIGURE 4:**
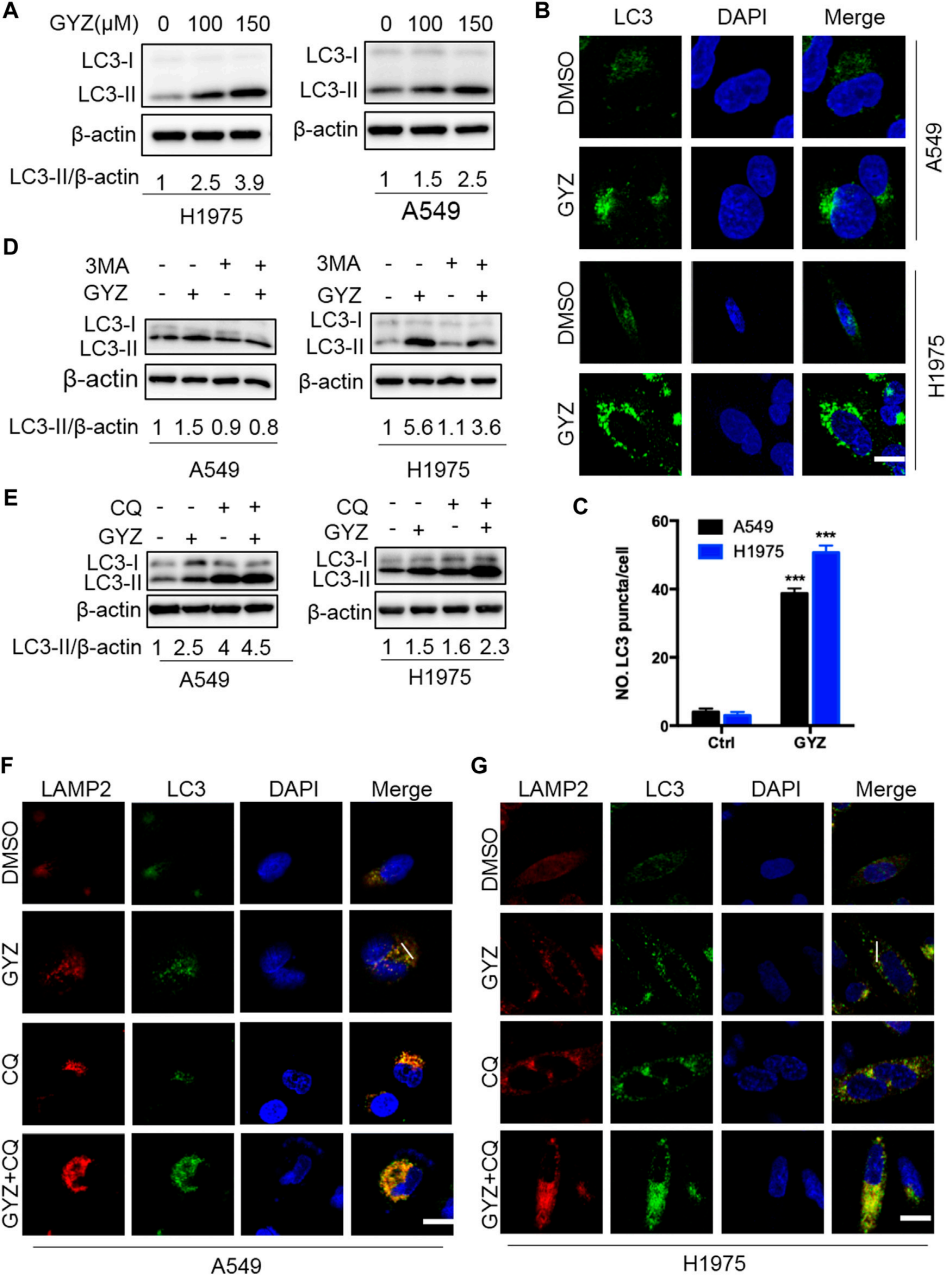
GYZ stimulates autophagy in NSCLC cells. **(A)** Immunoblot analysis of LC3 turnover expression in NSCLC cells treated with vehicle or GYZ for 24 h. **(B,C)** Immunofluorescent analysis of the accumulation of endogenous LC3 puncta in the absence or presence of GYZ (A549 150 μM, H1975 100 μM) for 24 h. Scale bars, 10 μm. **(D)** NSCLC cells were treated with vehicle, GYZ (A549 150 μM, H1975 100 μM), 3-MA (10 mM), or in combination for 24 h. Immunoblot analysis was used to examine LC3 turnover. **(E)** NSCLC cells were treated with GYZ (A549 150 μM, H1975 100 μM) with or without CQ (5 μM) for 24 h. LC3 turnover was detected by immunoblotting. **(F,G)** The colocalization of endogenous LC3 with LAMP2 was quantitated by immunofluorescent analysis in the treatment of GYZ (A549 150 μM, H1975 100 μM) with or without CQ (5 μM) for 24 h. Scale bars, 10 μm.

As accumulating reports have emphasized the potential application of drug-induced autophagy in antitumor therapies (Dou et al., 2016; Chen et al., 2019; Jiang et al., 2020), we investigated whether GYZ regulated autophagy in NSCLC cells. To verify this hypothesis, we first evaluated the protein expression levels of autophagy-related genes in GYZ-treated NSCLC cells. GYZ treatment resulted in significant autophagy induction as evidenced by increased conversion of LC3-I to lipidated LC3-II and the degradation of p62, the classical markers of autophagy, in a time- and dose-dependent manner in various NSCLC cells (Figure 4A and Supplementary Figures S1A,E). In agreement with this, the effect of GYZ on the formation of autophagosome membrane was further evidenced by the accumulation of endogenous LC3 puncta in GYZ-treated cells compared with control cells by immunofluorescent staining (Figures 4B,C). Moreover, treatment with 3MA, an inhibitor of class III PI3K, could restore the elevation of LC3-II levels in GYZ-treated cells (Figure 3D and Supplementary Figure S1B). Together, these findings indicate that GYZ promotes the initiation process of autophagy in NSCLC cells.

To figure out whether GYZ promotes complete autophagic flux, GYZ treatment accompanied with autolysosome inhibitor CQ was performed. The combinatorial treatment resulted in accumulation of LC3-II and enhanced endogenous LC3 puncta (Figures 4E–G and Supplementary Figure S1C). To further ascertain the fusion of lysosome with autophagosome in GYZ-treated NSCLC cells, we evaluated the colocalization of LC3 with the lysosome marker, LAMP2. GYZ treatment induced marked colocalization of LC3 with LAMP2, indicating the fusion of lysosome with autophagosome (Figures 4F,G and Supplementary Figures S2A,B). In agreement, we transfected a tandem mRFP-GFP tagged LC3 plasmid and found that combinatorial treatment of CQ with GYZ resulted in increased formation of yellow fluorescent autophagosomes (GFP^+^RFP^+^ signal), while GYZ-treated NSCLC cells displayed accumulation of red fluorescent autolysosomes (GFP^−^RFP^+^ signal) (Supplementary Figures S2C,D). Taken together, these data reveal that GYZ stimulates complete autophagic flux in NSCLC cells.

### Inhibition of Autophagy Augments the Antitumor Activity of GYZ in NSCLC Cells

**FIGURE 5:**
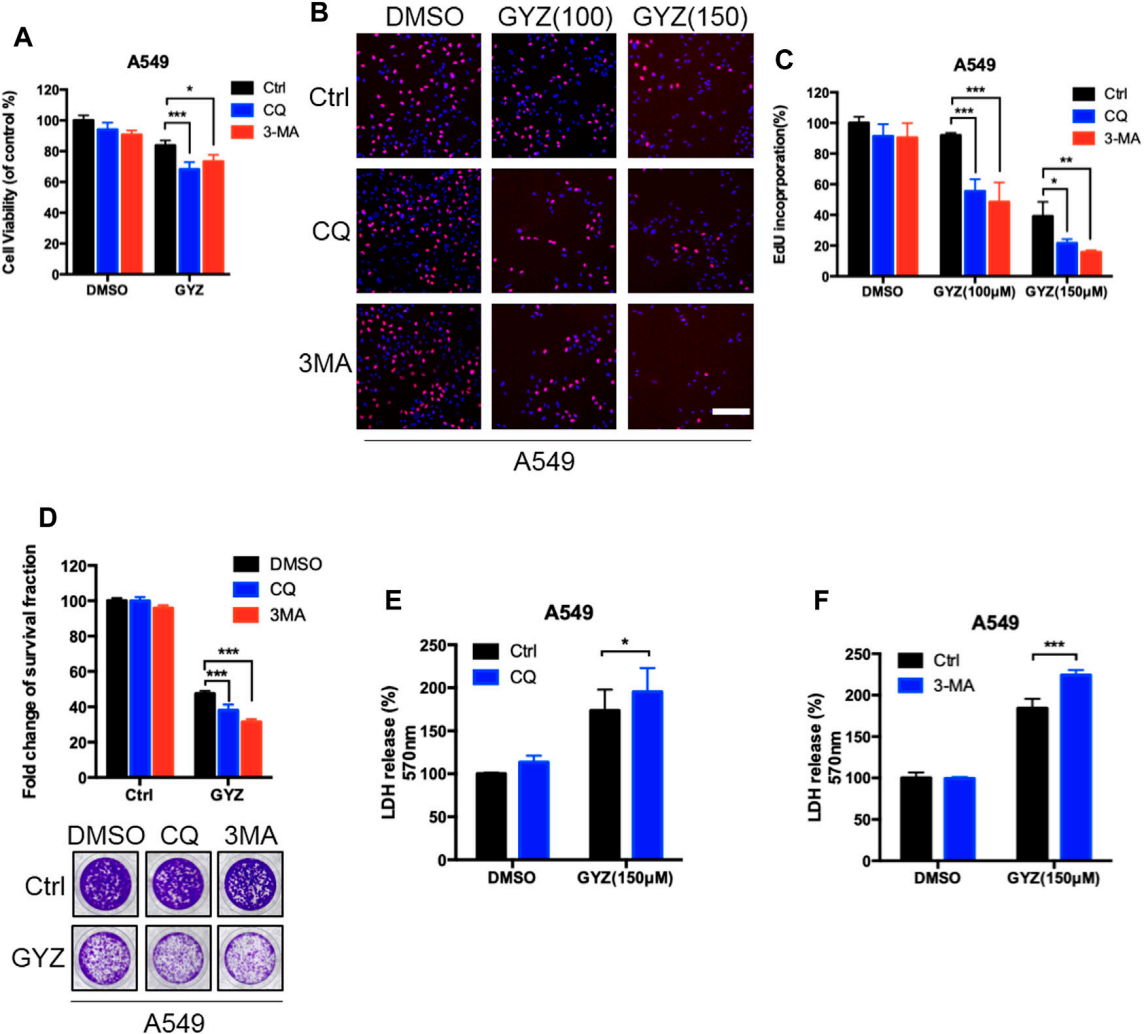
Inhibition of autophagy augments the antitumor activity of GYZ in NSCLC cells. **(A)** A549 cells were treated with 3-MA (10 mM) or CQ (5 μM) in the absence or presence of GYZ (100 μM) for 48 h. Cell growth was evaluated by MTT assay. **(B, C)** A549 Cells were treated with the indicated concentrations of GYZ in the absence or presence of 3-MA (10 mM) or CQ (5 μM). Cell proliferation was detected by EdU incorporation assay. **(D)** A549 cells were treated with CQ (5 μM) or 3-MA (10 mM) in the absence or presence of GYZ (150 μM) for 2 weeks. Cell proliferation was performed by colony-formation assay. E, F) A549 cells were treated with 3-MA (10 mM) or CQ (5 μM) in the absence or presence of GYZ (150 μM) for 48 h. The cytotoxicity was examined by the release of LDH. All data are means ± SD. *p < 0.05, **p < 0.01, ***p < 0.001.

To identify whether autophagy was involved in the anti-NSCLC effect of GYZ, combinational use of 3MA, CQ (both autophagy inhibitors) with GYZ were treated in NSCLC cells. As shown in Figure 5A and supplementary Figure S3A, an obvious decrease in cell proliferation was observed in GYZ-treated NSCLC cells in either combination with CQ or 3MA. Consistently, similar results were found by EdU labeling and colony formation analysis (Figures 5B–D and Supplementary Figures S3B–D). We also performed LDH release assay and found that either combinational use of 3MA or CQ with GYZ boosted GYZ-induced cytotoxicity in A549 and H1975 cells (Figures 5E,F and Supplementary Figures S3E,F). In conclusion, these data suggest that inhibition of autophagy augments the antitumor activity of GYZ in NSCLC cells.

### Inhibition of Akt/mTOR Signaling Is Responsible for GYZ-Induced Autophagy

**FIGURE 6:**
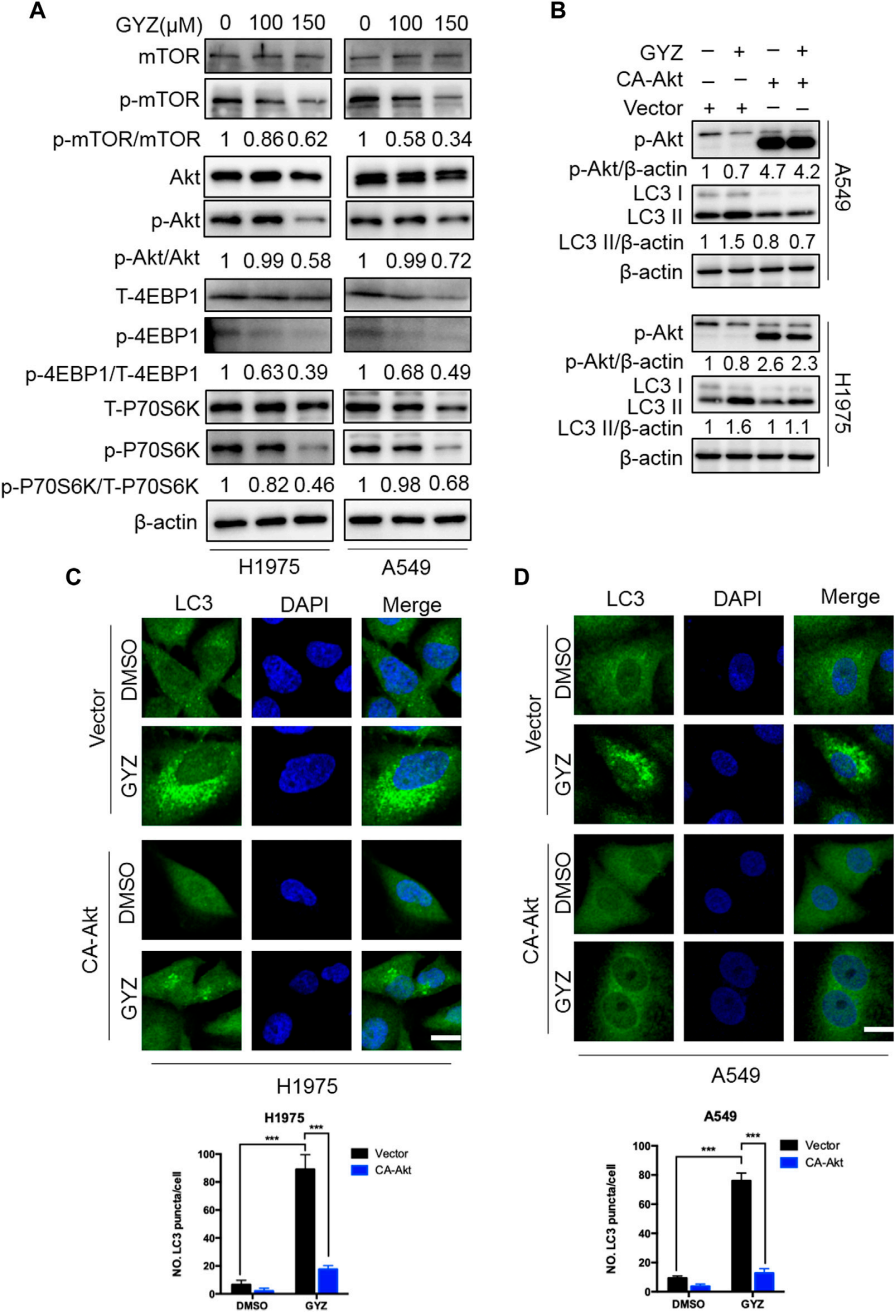
Inhibition of Akt/mTOR signaling is responsible for GYZ-induced autophagy. **(A)** Immunoblot analysis of phosphorylation of mTOR, Akt, 4EBP1 and p70S6K expression in NSCLC cells treated with the indicated concentrations of GYZ for 24 h. Total mTOR, Akt, 4EBP1, and p70S6K expression was examined as the internal control, respectively. **(B)** Cells were transfected with empty vectors (pECE) or with constitutively active CA-Akt plasmids for about 36 h, and then cells were treated with GYZ (150 μM) for another 24 h. Akt phosphorylation, and LC3 turnover were detected by immunoblotting. **(C,D)** H1975 cells **(C)** and A549 cells **(D)** were transfected with constitutively active CA-Akt plasmids or with empty vectors (pECE) for about 36 h in the absence or presence of GYZ (A549 150 μM, H1975 100 μM) for 24 h. Scale bars, 10 μm. All data are means ± SD. ***p < 0.001.

It has previously been shown that Akt/mTOR acts as a classical negative modulator of autophagy induced by antitumor drugs (Green and Levine, 2014; Dou et al., 2016; Zhou L. et al., 2019), we thus evaluate whether the Akt/mTOR pathway was inhibited in GYZ-treated NSCLC cells. As shown in Figure 6A, GYZ treatment obviously inhibited the Akt/mTOR pathway, as examined by decreased phosphorylation levels of mTOR, Akt, 4EBP1 and p70S6K. To further examine whether the Akt/mTOR pathway is involved in GYZ-induced autophagy, we transfected CA-Akt (a constitutively active form of Akt) plasmids into NSCLC cells in order to restore GYZ-induced Akt/mTOR inhibition. As shown in Figures 6B–D, LC3-II conversion and LC3 puncta accumulation were marked reduced within Akt reactivation in GYZ-treated cells. Furthermore, to figure out whether cytoprotective autophagy has strongly been linked to ROS, GYZ treatment accompanied with NAC was performed. As shown in Supplementary Figure S1D, we found that NAC attenuated guaiazulene-induced autophagy. Taken together, these findings suggest that the Akt/mTOR pathway is a key mediator of GYZ-induced autophagy in NSCLC cells.

### GYZ Exhibits Antitumor Effect Against NSCLC *In Vivo*

**FIGURE 7:**
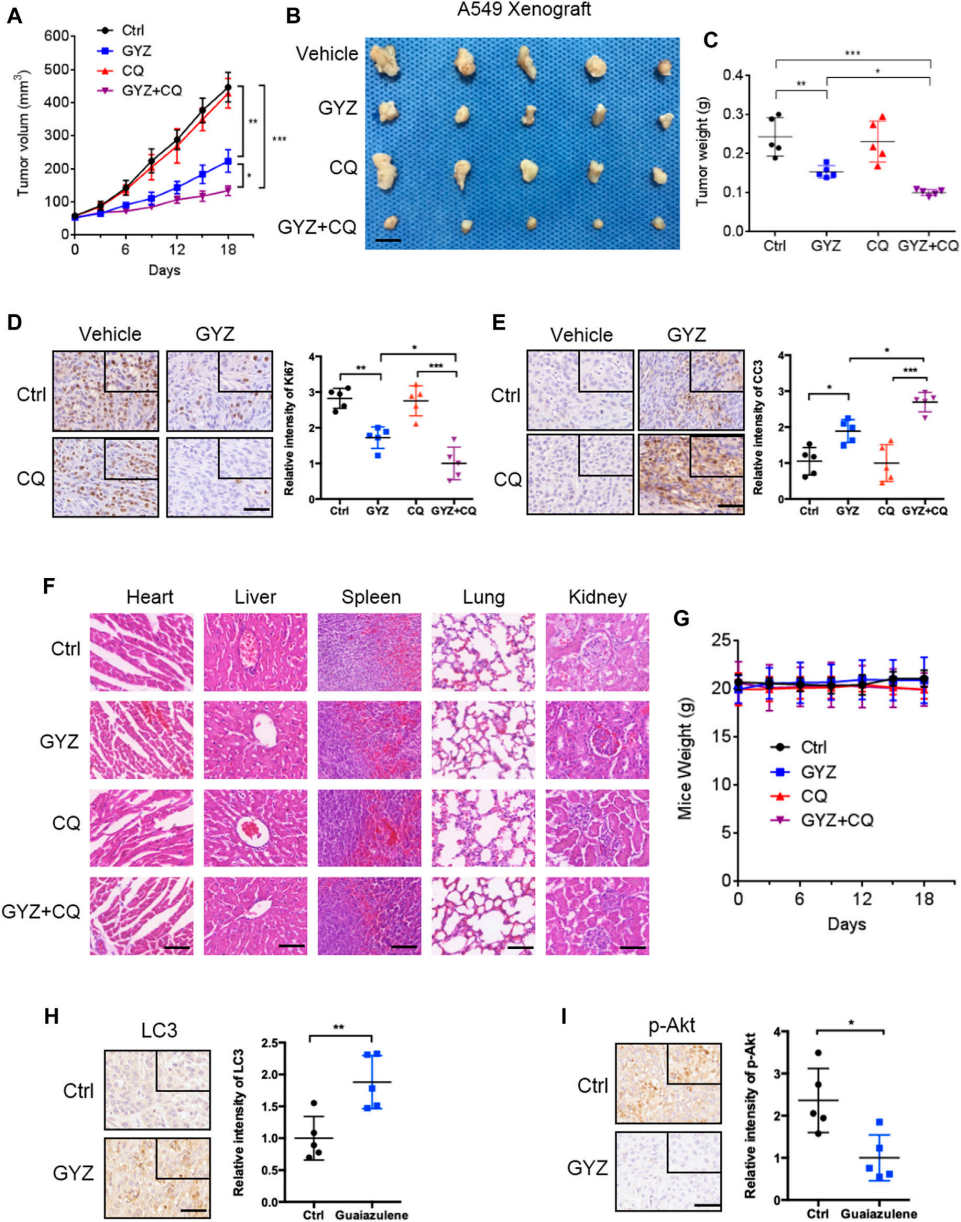
GYZ exhibits antitumor effect against NSCLC *in vivo*. **(A)** The volume of tumors from vehicle-, GYZ-, CQ-, or GYZ + CQ-treated mice (five mice per group) bearing A549 subcutaneous tumor xenografts was examined at the indicated time points. **(B)** The images of isolated tumors were shown. The isolated tumors were A549 subcutaneous tumor xenografts from vehicle-, GYZ-, CQ-, or GYZ+CQ-treated mice. Scale bars, 1 cm. **(C)** The weight of tumors from vehicle-, GYZ-, CQ-, or GYZ+CQ-treated group. **(D)** Immunohistochemistry analysis for Ki67 expression in tumor tissues from A549 xenografts treated with vehicle, GYZ (25 mg/kg/day), CQ (15 mg/kg/day), or GYZ+CQ. Scale bars, 50 μm. **(E)** Immunohistochemistry analysis for cleaved-caspase 3 expression in tumor tissues from A549 xenografts treated with vehicle, GYZ (25 mg/kg/day), CQ (15 mg/kg/day), or GYZ+CQ. Scale bars, 50 μm. F) Hematoxylin and eosin staining of the lung, liver, heart, spleen and kidney in mice treated with vehicle, GYZ (25 mg/kg/day), CQ (15 mg/kg/day), or GYZ+CQ. Scale bars, 100 μm. G) The weight of mice from vehicle-, GYZ-, CQ-, or GYZ + CQ-treated group. **(H,I)** Tumor tissues from A549 xenografts treated with vehicle or GYZ were assessed by immunohistochemistry analysis for LC3, and p-Akt expression. Scale bars, 50 μm. All data are means ± SD. *p < 0.05, **p < 0.01, ***p < 0.001.

To further prove the biologic effects of GYZ *in vivo*, we then generated a mouse xenograft model by subcutaneously inoculating the human NSCLC A549 cell line into nude mice. Consistent with *in vitro* study, xenografts treated with GYZ grew slower than the control group, as well the combinatorial treatment of GYZ with CQ compared with that of GYZ treatment alone (Figure 7A). As shown in Figures 7B,C, the anti-NSCLC effects of GYZ *in vivo* were also evidenced by the smaller size and lighter weight of tumors compared with those of the vehicle group. Notably, the combinatorial treatment of GYZ with CQ displayed a further decline in xenograft tumor weight and size compared with that of GYZ treatment alone. Moreover, immunohistochemical staining of Ki67 was performed to determine whether the proliferation status of tumor cells was changed. We found that xenografts treated with placebo displayed stronger Ki67 intensity than those treated with GYZ, and the combinatorial treatment of GYZ with CQ showed a further reduction of Ki67 staining than GYZ treatment alone (Figure 7D). Furthermore, obvious apoptosis was detected in tumors from GYZ-treated mice as evidenced by increased cleaved-caspase 3 staining, and the combinatorial treatment of GYZ with CQ showed a further increase of cleaved-caspase 3 intensity (Figure 7E). These data suggest that GYZ significantly inhibits the growth of NSCLC cells *in vivo* and combinatorial use of autophagy inhibitor further enhances the anti-NSCLC effect of GYZ. In addition, as shown in Figures 7E,F, the mice weight and pathologic features of major organs under GYZ treatment displayed no significant changes, indicating that GYZ has no obvious adverse effect or toxic in mice under the tested concentration. Furthermore, we also detected the expression levels of LC3 and p-Akt in tumor tissues from xenografts. Consistently, xenografts treated with GYZ performed stronger LC3 staining intensity and decreased phosphorylation levels of Akt (Figures 7H,I). Collectively, these results demonstrate that GYZ-induced autophagy is indeed through the suppression of Akt/mTOR pathway *in vivo*.

### GYZ Synergizes With PTX to Suppress the Growth of NSCLC Cells

**FIGURE 8:**
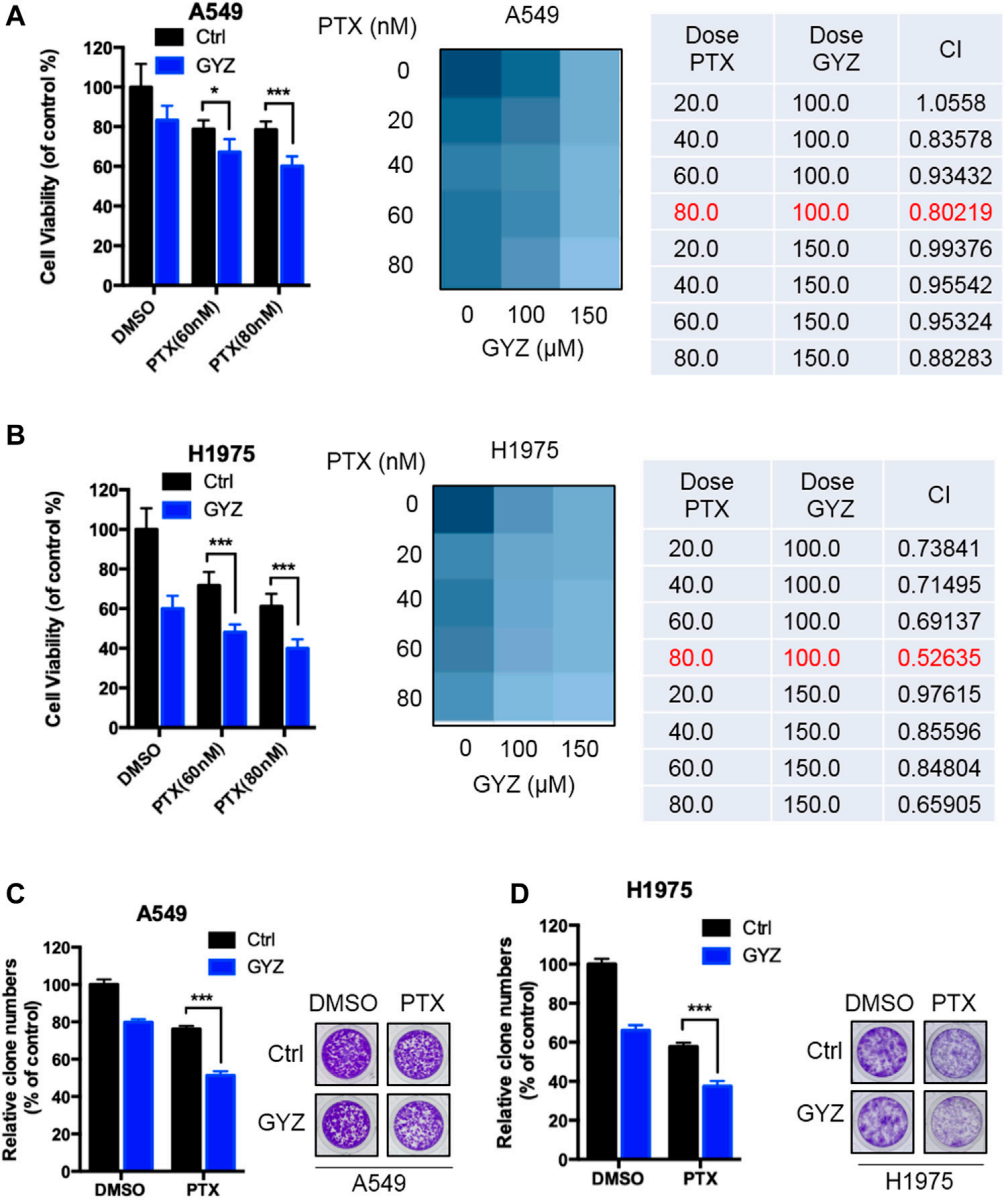
GYZ synergizes with PTX to suppress the growth of NSCLC cells. **(A,B)** A549 cells **(A)** and H1975 cells **(B)** were treated with paclitaxel (PTX) as the indicated doses in the absence or presence of GYZ (100 μM) for 48 h. Cell growth was evaluated by MTT assay. The combination index (CI) of two drugs was listed. **(C,D)** A549 cells **(C)** and H1975 cells **(D)** were treated with PTX as the indicated doses in the absence or presence of GYZ (100 μM) for 2 weeks. Cell proliferation was performed by colony-formation assay. All data are means ± SD. *p < 0.05, ***p < 0.001.

Paclitaxel (PTX), one of the most commonly chemotherapeutic drugs employed in clinical NSCLC treatment, has previously been reported to display acquired drug resistance partially due to the stimulation of autophagy (Chen and Shi, 2016; Lu et al., 2018; Zhan et al., 2018). Based on this situation, we determined the combined antitumor effects of GYZ and paclitaxel on the response of NSCLC cells. As shown in Figures 8A,B, combinational treatment of GYZ with paclitaxel obviously inhibited the cell proliferation compared with monotherapy. Through analyzing the combination index (CI) of the two drugs using Compusyn software, we found that co-treatment of 80 nM PTX with 100 μM GYZ shows the lowest CI value in both A549 and H1975 cells, indicating a better synergic effect of PTX and GYZ under this concentration. In addition, the NSCLC cell growth was markedly decreased in response to co-treatment GYZ with paclitaxel compared with monotherapy (Figures 8C,D). In conclusion, these results demonstrate that GYZ can effectively enhance paclitaxel sensitivity to NSCLC cells.

## Discussion

**FIGURE 9:**
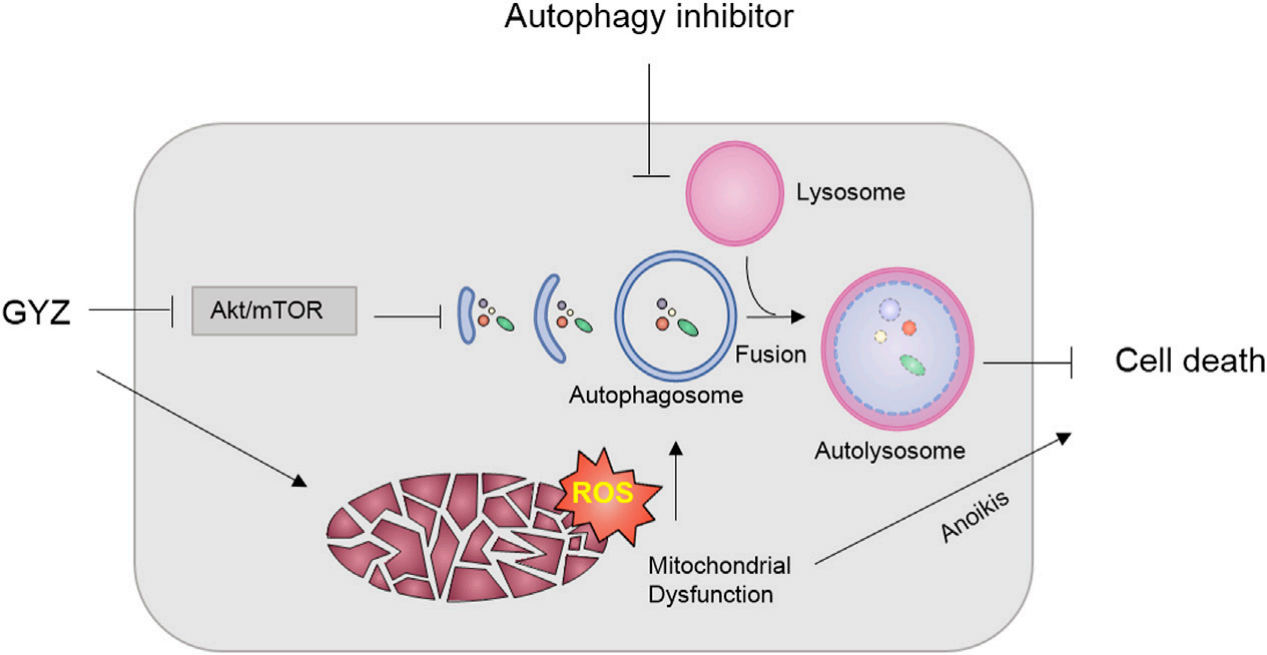
Schematic illustrating the mechanism of GYZ treatment accompanied with autophagy inhibition in NSCLC. On the one hand, GYZ induced mitochondrial dysfunction and increased the ROS level, thereby triggering anoikis in NSCLC; On the other hand, GYZ induced the formation of autophagosome and the fusion and degradation of autolysosome by blocking Akt/mTOR axis, resulting in cytoprotective autophagy and impaired anti-tumor effects of GYZ. Therefore, co-treatment with autophagy inhibitors could enhance the anti-tumor effects of GYZ in NSCLC.

GYZ, extracted from the oil of various plants, has been extended to multiple disease models (Vinholes et al., 2014a; Vinholes et al., 2014b). Increasing reports have proposed GYZ as a potential antitumor agent due to its remarkable effect on inhibition of various tumor growth (Yanagisawa et al., 1990; Kourounakis et al., 1997a; Guarrera et al., 2001; Wang et al., 2003). However, the efficacy of GYZ in the treatment of NSCLC and the underline molecular mechanisms are still elusive. In this study, our results first revealed that GYZ induced mitochondrial dysfunction, and thereby triggered anoikis in NSCLC. Meanwhile, GYZ suppressed Akt/mTOR signaling pathway, and thus activated cytoprotective autophagy. Accompanied with autophagy inhibitors would markedly enhance the anticancer effect of GYZ (Figure 9).

Over the several decades, GYZ was regarded as a strong antioxidant. For example, GYZ inhibited the metabolic activation of paracetamol by preventing cytochrome P450 activity due to its ability as a chain-breaking antioxidant (Kourounakis et al., 1997b). Furthermore, combinational use of 1 mM *tert*-BuOOH (one oxidant) with 1 mM GYZ could restore the cytotoxicity effect of *tert*-BuOOH on Caco-2 cells (Vinholes et al., 2014a). However, in recent years, studies have found that GYZ statistically enhanced total oxidative stress at concentrations nearly 100 μM comparing with control group in rat neuron cell line (Togar et al., 2015). In line with this, our present results demonstrate that GYZ increases cellular ROS levels and decreases ATP levels produced from mitochondria, leading to NSCLC anoikis. Thus, the precise role of GYZ on cellular redox homeostasis is context-dependent and the detailed mechanisms need further investigation.

In recent years, ROS-mediated signaling and oxidative stress have attracted much attention. It is important for cancer cells to obtain an optimal ROS level. On the one hand, cancer cells dependent on the ROS signaling for survival, proliferation and cell migration. On the other hand, if the levels of ROS exceed the threshold, ROS can induce various forms of cell death (Zhang et al., 2019c). For example, it has been shown that receptor-interacting protein kinase 3 (RIP3) activates the pyruvate dehydrogenase complex (PDC) to enhance aerobic respiration and induce mitochondrial ROS production. The accumulation of ROS then positively feeds back on tumor necrosis factor (TNF)-induced necroptosis (Yang et al., 2018). Another study revealed that in melanoma cells, iron stimulated ROS signaling initiated by carbonyl cyanide m-chlorophenyl hydrazone (CCCP). The accumulation of ROS results in the oxidation and oligomerization of Tom20, one of the mitochondrial outer membrane proteins. Then oxidized Tom20 recruited Bax to mitochondria, which facilitates the activation of caspase3 through cytochrome c release to cytosol, eventually triggering pyroptotic death by inducing gasdermin D (GSDMD) cleavage (Zhou et al., 2018). In this study, we found that GYZ stimulated mitochondrial ROS production and induced mitochondrial dysfunction, leading to anoikic cell death in NSCLC cells. Further studies are needed to investigate the mechanism of GYZ-induced ROS accumulation in NSCLC cells.

Anoikis is originally regarded as a physiologically relevant process in epithelial and endothelial cells which maintains development and tissue homeostasis (Frisch and Francis, 1994). Failure to regulate the anoikis program could lead to adherent cells proliferating at ectopic sites or surviving under suspension conditions. The deregulation of anoikis contributes to the formation of cancer and metastasis in distant organs (Frisch and Screaton, 2001; Gilmore, 2005). To date, lots of studies indicate that different types of integrins protect cells from anoikis and apoptosis via diverse signaling pathways. Their downstream molecules or pathways include FAK (Haun et al., 2018), Src kinase (Alanko et al., 2015), PI3K/Akt (Goundiam et al., 2012), mitogen activated protein kinase (MAPK) (Saranya et al., 2017) and integrin-linked kinase (ILK) (Attwell et al., 2000). FAK, one of the most classical integrin signaling molecules, is phosphorylated following integrin-mediated adhesion. Thus, activated FAK recruits and activates Src, consequently initialing the downstream cell survival signaling (Haun et al., 2018). Furthermore, PI3K is also one of the FAK-activated signaling proteins. Phosphorylated PI3K could recruit and activate its downstream molecule protein kinase B (PKB/Akt), therefore contributing to cell survival via several independent mechanisms (Paoli et al., 2013). In our study, we demonstrate that the protein level of Integrinβ3 and the phosphorylation level of Akt were obviously decreased after GYZ treatment in A549 and H1975 cells. But further studies are needed to explore the potential links between integrins and PI3K signaling in regulating GYZ-induced anoikis in NSCLC cells.

Autophagy is an essential catabolic process, which captures dysfunctional or damaged cellular components to fuse with lysosomes for degradation (Levine and Kroemer, 2019). Autophagy activation has been identified as a consequence of radiotherapy or chemotherapy according to studies on cancer treatment. However, the function of autophagy in tumor progression still remains controversial (Galluzzi et al., 2017). Throughout the past decade, our understanding of autophagy was to support tumor progression, which has nurtured distinct experimental approaches aimed at the improvement of autophagy inhibitors for tumor therapy (Zhou J. et al., 2019; Zhang et al., 2019b). However, over the past several years, intensive experimental efforts indicate that stimulation of autophagy might support the efficacy of anticancer regimens in malignant cells (Dou et al., 2016; Chen et al., 2019; Jiang et al., 2020). In this study, we found that GYZ promoted autophagosome formation and the fusion of autophagosomes and autolysosomes, leading to complete autophagic flux in NSCLC cells. We also first demonstrated that GYZ-induced autophagy displayed cytoprotective roles against GYZ-induced anoikis. It seems to be of particular interest to figure out the crosstalk between autophagy and the nature of anoikis in future cancer treatment.

Another key issue which should be addressed before the use of GYZ in clinic is the cytotoxic activity against human normal cells. Previous studies have demonstrated that guaiazulene and azulene derivatives were somewhat cytotoxic against normal human cells and displayed photomutagenic properties on bacterial strains (Fiori et al., 2011). Therefore, further efforts are needed to define the appropriate dose of guaiazulene for pursuing the balance between better anticancer efficacy and less side effects or new derivatives can also be developed to alleviate the side effects.

In summary, our study revealed that GYZ induced mitochondrial dysfunction and cellular ROS accumulation, thereby promoting anoikic cell death in NSCLC cells. Simultaneously, GYZ stimulated cytoprotective autophagy in NSCLC cells through suppression of the Akt/mTOR signaling pathway and co-treatment with autophagy inhibitors obviously enhanced the anti-NSCLC effects of GYZ. These findings provide profound insights into the antitumor efficacy of GYZ, which may provide a novel strategy for the treatment of NSCLC.

## Data Availability

The original contributions presented in the study are included in the article/Supplementary Material, further inquiries can be directed to the corresponding authors.

## References

[B1] Aggarwal C. Borghaei H. (2017). Treatment paradigms for advanced non‐small cell lung cancer at academic medical centers: involvement in clinical trial endpoint design. Oncol. 22, 700-708. 10.1634/theoncologist.2016-0345 PMC5469580 28408617

[B2] Alanko J. Mai A. Jacquemet G. Schauer K. Kaukonen R. Saari M. (2015). Integrin endosomal signalling suppresses anoikis. Nat. Cel Biol 17, 1412–1421. 10.1038/ncb3250 PMC4890650 26436690

[B3] Attwell S. Roskelley C. Dedhar S. (2000). The integrin-linked kinase (ILK) suppresses anoikis. Oncogene 19, 3811–3815. 10.1038/sj.onc.1203711 10949937

[B4] Bray F. Ferlay J. Soerjomataram I. Siegel R. L. Torre L. A. Jemal A. (2018). Global cancer statistics 2018: GLOBOCAN estimates of incidence and mortality worldwide for 36 cancers in 185 countries. CA: a Cancer J. clinicians 68, 394–424. 10.3322/caac.21492 30207593

[B5] Chanvorachote P. Pongrakhananon V. Halim H. (2015). Caveolin-1 regulates metastatic behaviors of anoikis resistant lung cancer cells. Mol. Cel Biochem 399, 291–302. 10.1007/s11010-014-2255-4 25351339

[B6] Chen K. Shi W. (2016). Autophagy regulates resistance of non-small cell lung cancer cells to paclitaxel. Tumor Biol. 37, 10539–10544. 10.1007/s13277-016-4929-x 26852748

[B7] Chen Y. Chen H.-N. Wang K. Zhang L. Huang Z. Liu J. (2019). Ketoconazole exacerbates mitophagy to induce apoptosis by downregulating cyclooxygenase-2 in hepatocellular carcinoma. J. Hepatol. 70, 66–77. 10.1016/j.jhep.2018.09.022 30287340

[B8] Chunhacha P. Pongrakhananon V. Rojanasakul Y. Chanvorachote P. (2012). Caveolin-1 regulates Mcl-1 stability and anoikis in lung carcinoma cells. Am. J. Physiology-Cell Physiol. 302, C1284–C1292. 10.1152/ajpcell.00318.2011 PMC3774262 22277751

[B9] Clara J. A. Monge C. Yang Y. Takebe N. (2019). Targeting signalling pathways and the immune microenvironment of cancer stem cells—a clinical update. Nat. Rev. Clin. Oncol. 17, 202–232. 10.1038/s41571-019-0293-2 31792354

[B10] Dou Q. Chen H.-N. Wang K. Yuan K. Lei Y. Li K. (2016). Ivermectin induces cytostatic autophagy by blocking the PAK1/Akt axis in breast cancer. Cancer Res. 76, 4457–4469. 10.1158/0008-5472.can-15-2887 27302166

[B11] Fiori J. Teti G. Gotti R. Mazzotti G. Falconi M. (2011). Cytotoxic activity of guaiazulene on gingival fibroblasts and the influence of light exposure on guaiazulene-induced cell death. Toxicol. Vitro 25, 64–72. 10.1016/j.tiv.2010.09.008 20854889

[B12] Frisch S. Francis H. (1994). Disruption of epithelial cell-matrix interactions induces apoptosis. J. Cel. Biol. 124, 619–626. 10.1083/jcb.124.4.619 PMC2119917 8106557

[B13] Frisch S. M. Ruoslahti E. (1997). Integrins and anoikis. Curr. Opin. Cel. Biol. 9, 701–706. 10.1016/s0955-0674(97)80124-x 9330874

[B14] Frisch S. M. Screaton R. A. (2001). Anoikis mechanisms. Curr. Opin. Cel. Biol. 13, 555–562. 10.1016/s0955-0674(00)00251-9 11544023

[B15] Galluzzi L. Bravo-San Pedro J. M. Demaria S. Formenti S. C. Kroemer G. (2017). Activating autophagy to potentiate immunogenic chemotherapy and radiation therapy. Nat. Rev. Clin. Oncol. 14, 247-258. 10.1038/nrclinonc.2016.183 27845767

[B16] Gewirtz D. A. (2014). The four faces of autophagy: implications for cancer therapy. Cancer Res. 74, 647–651. 10.1158/0008-5472.can-13-2966 24459182

[B17] Giancotti F. G. Ruoslahti E. (1999). Integrin signaling. science 285, 1028–1033. 10.1126/science.285.5430.1028 10446041

[B18] Gilmore A. P. (2005). Anoikis. Cell Death Differ 12, 1473–1477. 10.1038/sj.cdd.4401723 16247493

[B19] Goundiam O. Nagel M. D. Vayssade M. (2012). Akt and RhoA inhibition promotes anoikis of aggregated B16F10 melanoma cells. Cell. Biol. Int. 36, 311–319. 10.1042/cbi20110069 22070397

[B20] Green D. R. Levine B. (2014). To be or not to be? How selective autophagy and cell death govern cell fate. Cell 157, 65–75. 10.1016/j.cell.2014.02.049 24679527 PMC4020175

[B21] Guarrera M. Turbino L. Rebora A. (2001). The anti-inflammatory activity of azulene. J. Eur. Acad. Dermatol. Venerol 15, 486–487. 10.1046/j.1468-3083.2001.00340.x 11763400

[B22] Gunes T. Akin M. A. Sarici D. Hallac K. Kurtoglu S. Hashimoto T. (2013). Guaiazulene: a new treatment option for recalcitrant diaper dermatitis in NICU patients. J. Maternal-Fetal Neonatal Med. 26, 197–200. 10.3109/14767058.2012.722711 22928495

[B23] Haun F. Neumann S. Peintner L. Wieland K. Habicht J. Schwan C. (2018). Identification of a novel anoikis signalling pathway using the fungal virulence factor gliotoxin. Nat. Commun. 9, 1–14. 10.1038/s41467-018-05850-w 30166526 PMC6117259

[B24] Jiang J. Zhang L. Chen H. Lei Y. Zhang T. Wang Y. (2020). Regorafenib induces lethal autophagy arrest by stabilizing PSAT1 in glioblastoma. Autophagy 16, 106–122. 10.1080/15548627.2019.1598752 30909789 PMC6984601

[B25] Jiang L. Kon N. Li T. Wang S.-J. Su T. Hibshoosh H. (2015). Ferroptosis as a p53-mediated activity during tumour suppression. Nature 520, 57–62. 10.1038/nature14344 25799988 PMC4455927

[B26] Jin P. Jiang J. Xie N. Zhou L. Huang Z. Zhang L. (2019). MCT1 relieves osimertinib-induced CRC suppression by promoting autophagy through the LKB1/AMPK signaling. Cel Death Dis. 10, 1–15. 10.1038/s41419-019-1844-2 PMC6692318 31409796

[B27] Kourounakis A. P. Rekka E. A. Kourounakis P. N. (1997a). Antioxidant activity of guaiazulene and protection against paracetamol hepatotoxicity in rats. J. Pharm. Pharmacol. 49, 938–942. 10.1111/j.2042-7158.1997.tb06140.x 9306266

[B28] Kourounakis A. P. Rekka E. A. Kourounakis P. N. (1997b). Effect of guaiazulene on some cytochrome P450 activities. Implication in the metabolic activation and hepatotoxicity of paracetamol. Arch. Pharm. Pharm. Med. Chem. 330, 7–11. 10.1002/ardp.19973300103 9112807

[B29] Kumar P. Yadav A. Patel S. N. Islam M. Pan Q. Merajver S. D. (2010). Tetrathiomolybdate inhibits head and neck cancer metastasis by decreasing tumor cell motility, invasiveness and by promoting tumor cell anoikis. Mol. Cancer 9, 1–11. 10.1186/1476-4598-9-206 20682068 PMC2922193

[B30] Levine B. Kroemer G. (2019). Biological functions of autophagy genes: a disease perspective. Cell 176, 11–42. 10.1016/j.cell.2018.09.048 30633901 PMC6347410

[B31] Li S. Chen Y. Zhang Y. Jiang X. Jiang Y. Qin X. (2019). Shear stress promotes anoikis resistance of cancer cells via caveolin-1-dependent extrinsic and intrinsic apoptotic pathways. J. Cel Physiol 234, 3730–3743. 10.1002/jcp.27149 30171601

[B32] Lu X. Zhou D. Hou B. Liu Q.-X. Chen Q. Deng X.-F. (2018). Dichloroacetate enhances the antitumor efficacy of chemotherapeutic agents via inhibiting autophagy in non-small-cell lung cancer. Cmar 10, 1231-1241. 10.2147/cmar.s156530 PMC5962308 29844702

[B33] Paoli P. Giannoni E. Chiarugi P. (2013). Anoikis molecular pathways and its role in cancer progression. Biochim. Biophys. Acta (Bba) - Mol. Cel Res. 1833, 3481–3498. 10.1016/j.bbamcr.2013.06.026 23830918

[B34] Pongrakhananon V. Nimmannit U. Luanpitpong S. Rojanasakul Y. Chanvorachote P. (2010). Curcumin sensitizes non-small cell lung cancer cell anoikis through reactive oxygen species-mediated Bcl-2 downregulation. Apoptosis 15, 574–585. 10.1007/s10495-010-0461-4 20127174

[B35] Prateep A. Sumkhemthong S. Karnsomwan W. De-Eknamkul W. Chamni S. Chanvorachote P. (2018). Avicequinone B sensitizes anoikis in human lung cancer cells. J. Biomed. Sci. 25, 32. 10.1186/s12929-018-0435-3 29631569 PMC5890350

[B36] Pratsinis H. Haroutounian S. (2002). Synthesis and antioxidant activity of 3-substituted guaiazulene derivatives. Nat. Product. Lett. 16, 201–205. 10.1080/10575630290013585 12049221

[B37] Pritchard D. M. Watson A. J. M. (1996). Apoptosis and gastrointestinal pharmacology. Pharmacol. Ther. 72, 149–169. 10.1016/s0163-7258(96)00102-7 8981574

[B38] Qiao L. Wong B. C. Y. (2009). Targeting apoptosis as an approach for gastrointestinal cancer therapy. Drug Resist. updates 12, 55–64. 10.1016/j.drup.2009.02.002 19278896

[B39] Ramalingam S. S. Vansteenkiste J. Planchard D. Cho B. C. Gray J. E. Ohe Y. (2020). Overall survival with osimertinib in untreated, EGFR-mutated advanced NSCLC. N. Engl. J. Med. 382, 41–50. 10.1056/nejmoa1913662 31751012

[B40] Ravid D. Maor S. Werner H. Liscovitch M. (2005). Caveolin-1 inhibits cell detachment-induced p53 activation and anoikis by upregulation of insulin-like growth factor-I receptors and signaling. Oncogene 24, 1338–1347. 10.1038/sj.onc.1208337 15592498

[B41] Saranya J. Shilpa G. Raghu K. G. Priya S. (2017). Morus alba leaf lectin (MLL) sensitizes MCF-7 cells to anoikis by inhibiting fibronectin mediated integrin-FAK signaling through ras and activation of P38 MAPK. Front. Pharmacol. 8, 34. 10.3389/fphar.2017.00034 28223935 PMC5293820

[B42] Sarniak A. Lipińska J. Tytman K. Lipińska S. (2016). Endogenous mechanisms of reactive oxygen species (ROS) generation. Postepy Hig Med. Dosw 70, 1150–1165. 10.5604/17322693.1224259 27892899

[B43] Shaw A. T. Solomon B. J. Chiari R. Riely G. J. Besse B. Soo R. A. (2019). Lorlatinib in advanced ROS1-positive non-small-cell lung cancer: a multicentre, open-label, single-arm, phase 1-2 trial. Lancet Oncol. 20, 1691–1701. 10.1016/s1470-2045(19)30655-2 31669155

[B44] Togar B. Turkez H. Hacimuftuoglu A. Tatar A. Geyikoglu F. (2015). Guaiazulene: biochemical activity and cytotoxic and genotoxic effects on rat neuron and N2a neuroblastom cells. J. Intercult Ethnopharmacol 4, 29-33. 10.5455/jice.20141124062203 26401381 PMC4566767

[B45] Uehara M. Minemura H. Ohno T. Hashimoto M. Wakabayashi H. Okudaira N. (2018). *In vitro* antitumor activity of alkylaminoguaiazulenes. In Vivo 32, 541–547. 10.21873/invivo.11273 29695558 PMC6000785

[B46] Vachon P. H. (2018). Methods for assessing apoptosis and anoikis in normal intestine/colon and colorectal cancer. Methods Mol. Biol., 1765, 99–137. 10.1007/978-1-4939-7765-9_7 29589304

[B47] Vachon P. H. (2011). Integrin signaling, cell survival, and anoikis: distinctions, differences, and differentiation. J. Signal. Transduct 2011, 738137. 10.1155/2011/738137 21785723 PMC3139189

[B48] Vinholes J. Gonçalves P. Martel F. Coimbra M. A. Rocha S. M. (2014a). Assessment of the antioxidant and antiproliferative effects of sesquiterpenic compounds in *in vitro* Caco-2 cell models. Food Chem. 156, 204–211. 10.1016/j.foodchem.2014.01.106 24629959

[B49] Vinholes J. Rudnitskaya A. Gonçalves P. Martel F. Coimbra M. A. Rocha S. M. (2014b). Hepatoprotection of sesquiterpenoids: a quantitative structure-activity relationship (QSAR) approach. Food Chem. 146, 78–84. 10.1016/j.foodchem.2013.09.039 24176316

[B50] Wakabayashi H. Hashiba K. Yokoyama K. Hashimoto K. Kikuchi H. Nishikawa H. (2003). Cytotoxic activity of azulenes against human oral tumor cell lines. Anticancer Res. 23, 4747–4755. 14981922

[B51] Wang L. Yan J. Fu P. P. Parekh K. A. Yu H. (2003). Photomutagenicity of cosmetic ingredient chemicals azulene and guaiazulene. Mutat. Research/Fundamental Mol. Mech. Mutagenesis 530, 19–26. 10.1016/s0027-5107(03)00131-3 PMC3767376 14563527

[B52] Yanagisawa T. Kosakai K. Tomiyama T. Yasunami M. Takase K. (1990). Studies on anti-ulcer agents. II. Synthesis and anti-ulcer activities of 6-isopropylazulene-1-sodium sulfonate derivatives. Chem. Pharm. Bull. 38, 3355–3358. 10.1248/cpb.38.3355 1965495

[B53] Yang Z. Wang Y. Zhang Y. He X. Zhong C.-Q. Ni H. (2018). RIP3 targets pyruvate dehydrogenase complex to increase aerobic respiration in TNF-induced necroptosis. Nat. Cel Biol 20, 186–197. 10.1038/s41556-017-0022-y 29358703

[B54] Yu N. Xiong Y. Wang C. (2017). Bu-Zhong-Yi-Qi decoction, the water extract of Chinese traditional herbal medicine, enhances cisplatin cytotoxicity in A549/DDP cells through induction of apoptosis and autophagy. Biomed. Res. Int. 2017, 3692797. 10.1155/2017/3692797 28154825 PMC5244006

[B55] Zhan Y. Wang K. Li Q. Zou Y. Chen B. Gong Q. (2018). The novel autophagy inhibitor alpha-hederin promoted paclitaxel cytotoxicity by increasing reactive oxygen species accumulation in non-small cell lung cancer cells. Ijms 19, 3221. 10.3390/ijms19103221 PMC6214018 30340379

[B56] Zhang R. Tao F. Ruan S. Hu M. Hu Y. Fang Z. (2019a). The TGFβ1-FOXM1-HMGA1-TGFβ1 positive feedback loop increases the cisplatin resistance of non-small cell lung cancer by inducing G6PD expression. Am. J. Transl Res. 11, 6860-6876. 31814893 PMC6895501

[B57] Zhang Z. Gao W. Zhou L. Chen Y. Qin S. Zhang L. (2019b). Repurposing brigatinib for the treatment of colorectal cancer based on inhibition of ER-phagy. Theranostics 9, 4878-4892. 10.7150/thno.36254 31410188 PMC6691391

[B58] Zhang Z. Zhang L. Zhou L. Lei Y. Zhang Y. Huang C. (2019c). Redox signaling and unfolded protein response coordinate cell fate decisions under ER stress. Redox Biol. 25, 101047. 10.1016/j.redox.2018.11.005 30470534 PMC6859529

[B59] Zhou B. Zhang J.-Y. Liu X.-S. Chen H.-Z. Ai Y.-L. Cheng K. (2018). Tom20 senses iron-activated ROS signaling to promote melanoma cell pyroptosis. Cell Res 28, 1171–1185. 10.1038/s41422-018-0090-y 30287942 PMC6274649

[B60] Zhou H. Xu M. Gao Y. Deng Z. Cao H. Zhang W. (2014). Matrine induces caspase-independent program cell death in hepatocellular carcinoma through bid-mediated nuclear translocation of apoptosis inducing factor. Mol. Cancer 13, 1–11. 10.1186/1476-4598-13-59 24628719 PMC4007561

[B61] Zhou J. Zhang L. Wang M. Zhou L. Feng X. Yu L. (2019a). CPX targeting DJ-1 triggers ROS-induced cell death and protective autophagy in colorectal cancer. Theranostics 9, 5577-5594. 10.7150/thno.34663 31534504 PMC6735393

[B62] Zhou L. Gao W. Wang K. Huang Z. Zhang L. Zhang Z. (2019b). Brefeldin A inhibits colorectal cancer growth by triggering Bip/Akt‐regulated autophagy. FASEB j. 33, 5520–5534. 10.1096/fj.201801983r 30668917

