# Supplementary material for: Guaiazulene Triggers ROS-Induced Apoptosis and Protective Autophagy in Non-small Cell Lung Cancer
Source: Front Pharmacol. 2021 Apr 15;12:621181. doi: 10.3389/fphar.2021.621181 (PMC8082441; doi:10.3389/fphar.2021.621181)
Supplement: Supplementary file 1 [file datasheet1.pdf]

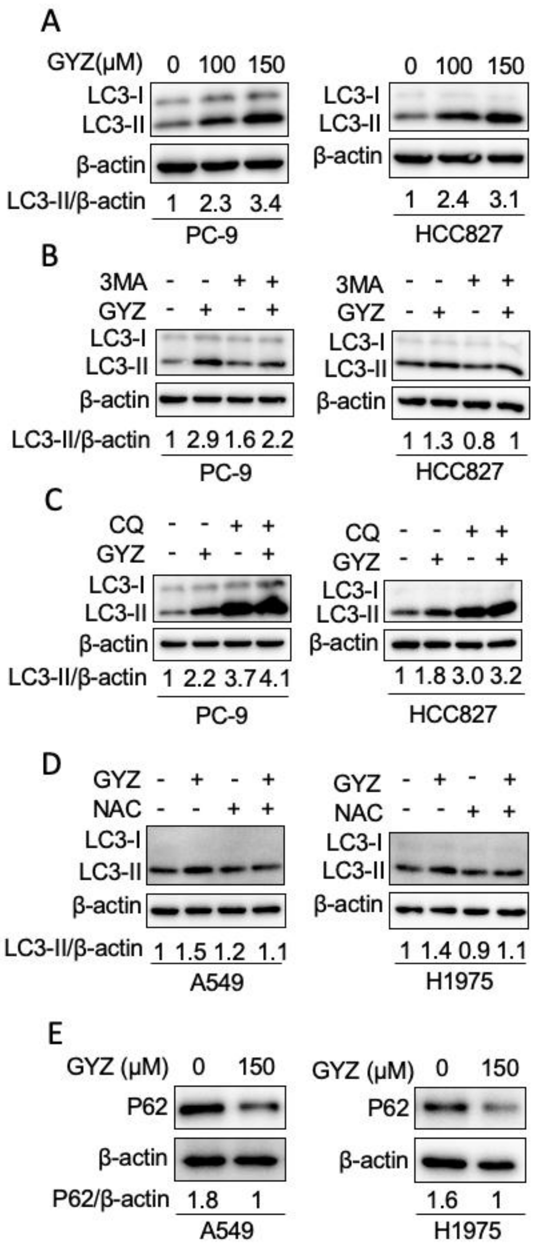

Supplementary figure 1, GYZ stimulate autophagy in NSCLC cells. A) Immunoblot analysis of LC3 turnover expression in NSCLC cells treated with vehicle or GYZ for 24 h. B) NSCLC cells were treated with vehicle, GYZ (100µM), 3-MA (10mM), or in combination for 24 h. Immunoblot analysis was used to examine LC3 turnover. C) NSCLC cells were treated with GYZ (100µM) with or without CQ (5µM) for 24 h. LC3 turnover was detected by immunoblotting. D) NSCLC cells were treated with GYZ (150µM) with or without NAC (2mM) for 24 h. LC3 turnover was detected by immunoblotting. E) Immunoblot analysis of P62 expression in NSCLC cells treated with vehicle or GYZ for 24 h.

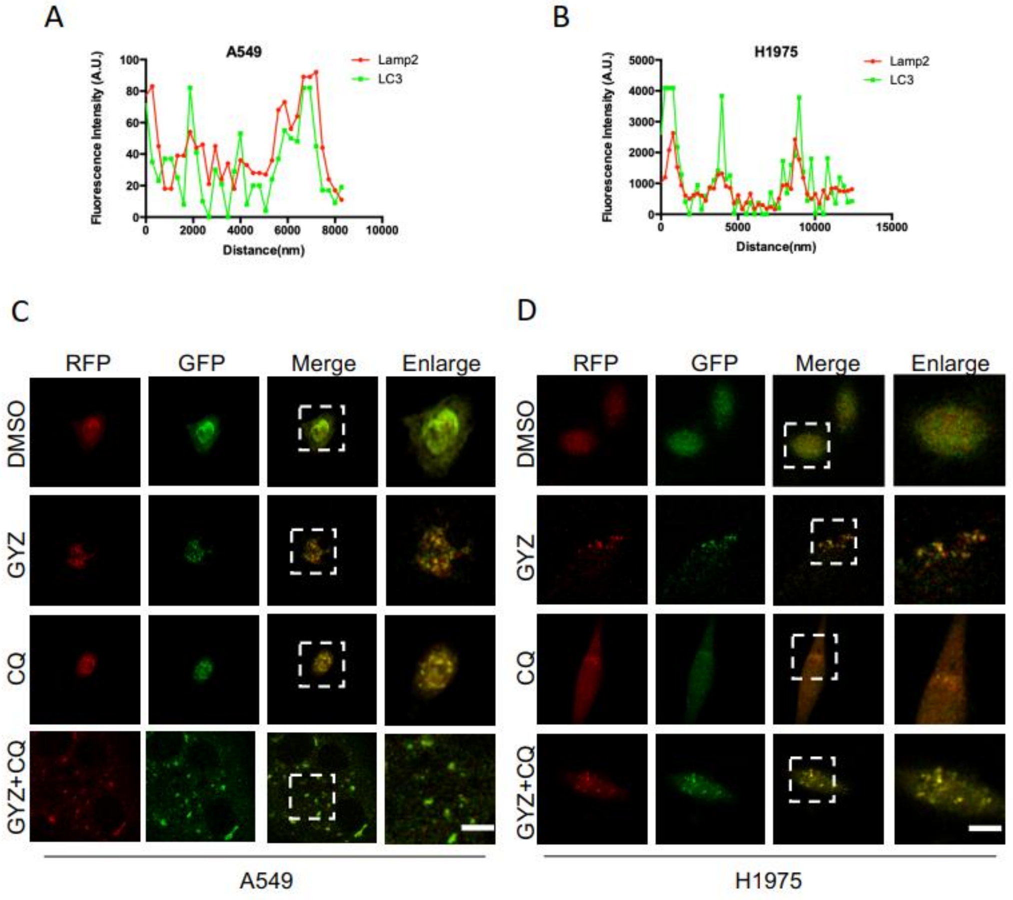

Supplementary figure 2, GYZ stimulate autophagy in NSCLC cells. A, B) The colocalization of endogenous LC3 with LAMP2 was quantitated by immunofluorescent analysis in the treatment of GYZ (A549 150μM, H1975 100μM) with or without CQ (5μM) for 24 h. C, D) Cells transiently transfected with tandem mRFP-GFP-tagged LC3 were performed by immunofluorescence analysis in the treatment of GYZ (A549 150μM, H1975 100μM) with or without CQ (5μM) for 24 h. Scale bars, 10 mm.

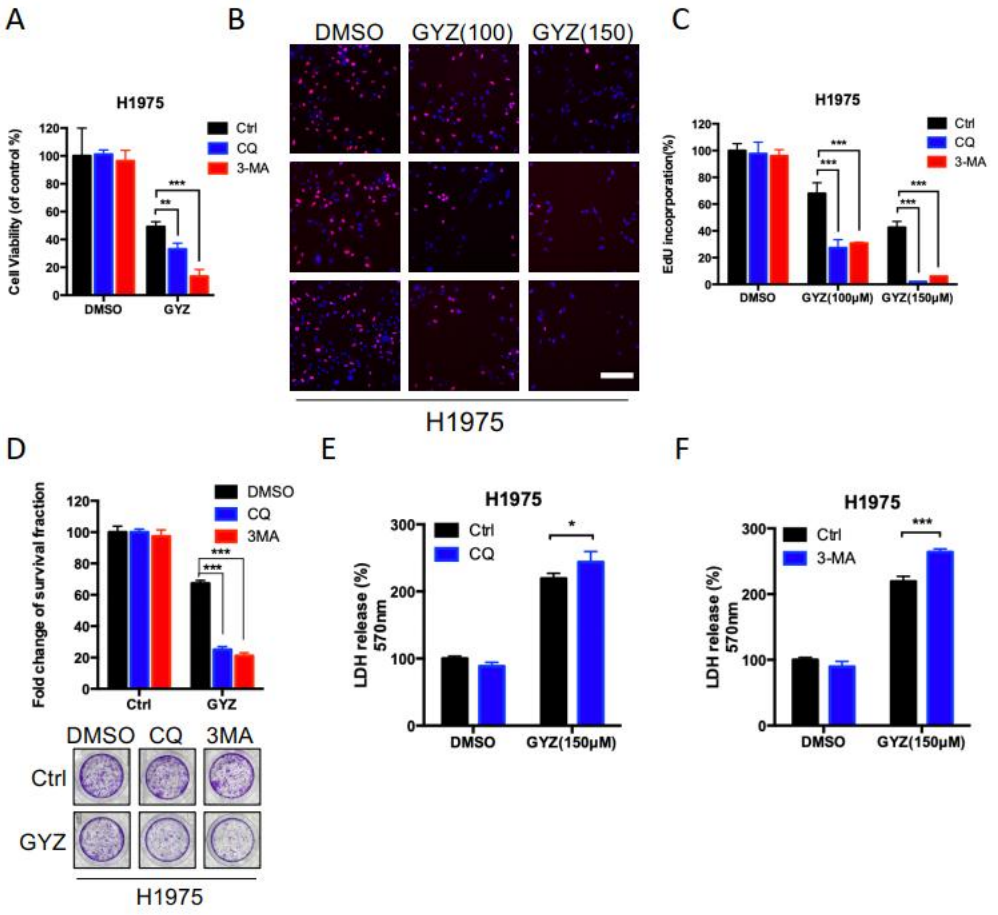

Supplementary figure 3. Inhibition of autophagy augments the antitumor activity of GYZ in NSCLC cells. A) H1975 cells were treated with 3-MA (10mM) or CQ (5μM) in the absence or presence of GYZ (100μM) for 48 h. Cell growth was evaluated by MTT assay. B, C) H1975 cells were treated with the indicated concentrations of GYZ in the absence or presence of 3-MA (10mM) or CQ (5μM). Cell proliferation was detected by EdU incorporation assay. D) H1975 cells were treated with CQ (5μM) or 3-MA (10mM) in the absence or presence of GYZ (100μM) for 2 wk. Cell proliferation was performed by colony-formation assay. E, F) H1975 cells were treated with 3-MA (10mM) or CQ (5μM) in the absence or presence of GYZ (150μM) for 48 h. The cytotoxicity was examined by the release of LDH. All data are means ± SD. **P<0.05, **P<0.01, ***P<0.001*.
